# Acupuncture combined with mirror therapy for post-stroke dyskinesia: A meta-analysis and systematic review

**DOI:** 10.1097/MD.0000000000038733

**Published:** 2024-06-28

**Authors:** Yufeng Peng, Nan Li, Xiaona Du, Guanghui Zhang, Shouqiang Huang, Jiao Ma

**Affiliations:** a Acupuncture Department, Ningbo Zhenhai Hospital of Traditional Chinese Medicine, Ningbo, China; b Clinical Skill Training Center, The Second Affiliated Hospital Zhejiang University School of Medicine, Hangzhou, China; c School of Medical Technology and Information Engineering, Zhejiang Chinese Medical University, Hangzhou, China; d Department of Emergency Medicine, Taihe Hospital, Hubei, China.

**Keywords:** acupuncture, meta-analysis, mirror therapy, movement disorders, stroke

## Abstract

**Background:**

Dyskinesia is one of the most common complications of stroke. Acupuncture therapy (AT) and mirror therapy (MT) are promising rehabilitation measures for the treatment of post-stroke dyskinesia. Although some studies suggested that AT and MT are effective and safe for dyskinesia, the effects, and safety remain uncertain due to lacking strong evidence. The purpose of this study is to investigate the efficacy and safety of AT combined with MT in the treatment of post-stroke dyskinesia.

**Methods:**

We searched the following databases: PubMed, Web of Science, Cochrane Library, EMBASE, Medline, China Knowledge Network, WANFANG, and China Biomedical Literature Database, from inception to 1 January 2023 to identify eligible studies. Total effective rate, the Fugl–Meyer assessment scale (FMA) upper and lower limb scores, modified Barthel index scores, Berg balance scale, modified Ashworth scale, and adverse reactions were adopted as outcome indicators. The Grading of Recommendations Assessment Development and Evaluation system was used by 2 independent reviewers to assess the quality of evidence for the outcome indicators included in the study. The statistical analysis was conducted by RevMan V.5.4 software.

**Results:**

A total of 24 randomized controlled studies included 2133 patients with post-stroke dyskinesia were included. The total effective rate of AT combined with MT was more advantageous in the treatment of post-stroke dyskinesia (relative risk = 1.31, 95% confidence interval [CI] [1.22–1.42], *Z* = 6.96, *P* < .0001). AT combined with MT was more advantageous for FMA upper limb score (mean difference [MD] = 6.67, 95% CI [5.21–8.13], *Z* = 8.97, *P* < .00001) and FMA lower limb score (MD = 3.72, 95% CI [2.81–4.63], *Z* = 7.98, *P* < .00001). Meta-analysis showed that AT combined with MT for post-stroke dyskinesia had a more advantageous modified Barthel index score (MD = 9.51, 95% CI [7.44–11.58], *Z* = 9.01, *P* < .00001).

**Conclusion:**

AT combined with MT is effective in improving motor function and daily living ability of patients, especially in improving muscle spasms. However, these results should be regarded with caution given the low quality of evidence for the evaluation results.

## 1. Introduction

Stroke, commonly known as a cerebrovascular accident, is an injury to the brain caused by either the blockage or rupture of blood vessels. This disrupts the circulation of blood within the brain, potentially leading to high rates of both incidence and disability. It is estimated that approximately 70 to 80% of stroke survivors suffer from various degrees of sequelae, among which dyskinesia is prevalent.^[1]^ Post-stroke dyskinesia, a form of central paralysis, results from damage to the pyramidal tract with typical features of abnormal muscle activity, notably in the flexors of the upper limbs and the extensors of the lower limbs. Clinically, it presents as hypokinesia, spasticity, restricted limb motion, and either diminished or complete loss of voluntary movement on one side of the body. These symptoms significantly affect the patients’ quality of life.^[2]^

Currently, the functional rehabilitation methods for post-stroke dyskinesia primarily include pharmacotherapy, assistive technologies, rehabilitation physiotherapy, and surgical interventions. In the acute phase of recovery, clinical practice often favors a combination of medication and rehabilitation. Common medications include aspirin, simvastatin, clopidogrel, warfarin, and other foundational treatments aimed at blood pressure control, anticoagulation, and lipid stabilization. For managing dyskinesia during the post-stroke spastic phase, skeletal muscle relaxants are frequently utilized as supplementary medications. Additionally, recent studies have also shown that selective 5-hydroxytryptamine reuptake inhibitors^[3]^ can provide some improvement in post-stroke dyskinesia. However, according to the latest American heart association/American stroke association guidelines,^[4]^ there is no good drug cure for the recovery of motor function after stroke, and some drugs have many adverse reactions after long-term use. Surgical treatment includes a variety of fasciolysis and neurectomy, but it is invasive and easy to destroy the nerve pathway, which is not the first choice in clinical practice.

Mirror therapy (MT),^[5]^ a combined rehabilitation therapy based on visual feedback, visual illusion, and virtual reality, can promote bilateral hemispheric balance in the brain and improve post-stroke dyskinesia^[6]^ by modulating brain neural networks through motor imagery. MT, proposed by Ramachandran,^[7]^ is not only effective in treating stroke patients with hemiplegia,^[6,8]^ but is also easy to implement and has no side effects.^[9,10]^ A randomized controlled trial (RCT) on upper limb movement disorders after stroke^[11]^ showed that the MT group was significantly better than the control group in improving elbow flexion, wrist flexion, wrist extension, and finger flexion and extension.

Acupuncture therapy (AT) as a traditional external treatment method in Chinese medicine, has an effect on opening meridians, moving qi and blood, and regulating yin and yang, which is now widely used in clinical practice to treat patients with post-stroke hemiplegia. Modern medicine has proved that acupuncture can dilate blood vessels, improve blood circulation in the brain, and enhance blood oxygen supply of injured neural tissues, so as to reduce the formation of free radicals, protect the formation and growth of neurons and synapsis, and promote neural stem cell proliferation and neurological function repair.^[12]^ Therefore, AT is conducive to improving patients’ limb motor functions and improving their quality of life.^[13]^ Thus, acupuncture has an important role in clinical motor function rehabilitation.^[14]^

Rehabilitating post-stroke dyskinesia is challenging. Relying solely on a single rehabilitation method often falls short of optimal therapeutic outcomes. Consequently, comprehensive intervention remains a necessity in clinical settings. Both AT and MT are characterized by their safety, convenience, efficacy, affordability, and lack of side effects. Compared with the potential drug resistance in drug therapy and the high cost of device-based rehabilitation, the combined approach of AT and MT is a more appropriate choice. There have been many reports of AT combined with MT in the treatment of post-stroke dyskinesia. These studies consistently demonstrate that such a combined approach significantly enhances upper and lower limb motor functions, improves activities of daily living, and alleviates muscle spasticity in stroke survivors, with better effects than using MT or AT alone. Nevertheless, no relevant meta-analysis has been conducted to provide higher evidence-based medical evidence for this combination therapy.

As a quantitative statistical method widely used in clinical evidence-based research, meta-analysis contributes to further guiding medical research and developing clinical guidelines and is of great significance to clinical research. Therefore, this study scientifically evaluates the efficacy and safety of AT combined with MT in the treatment of post-stroke dyskinesia through meta-analysis, so as to provide more powerful evidence for clinical treatment.

## 2. Methods

### 2.1. Registration and protocol

This study was completed in accordance with the Preferred Reporting Initiative for Systematic Reviews and Meta-Analysis Project guidelines.^[15]^ This systematic review program was registered on the INPLASY website. Registration number: INPLASY2022100100. Since this study is on the basis of published studies, ethical approval is not required.

### 2.2. Literature search strategy

This study searched for literature in relevant electronic databases, including Cochrane, Embase, Web of Science, Pubmed, China Knowledge Network Database, WanFang Database (WanFang), Chongqing Vipers Database (VIP), and China Biomedical Literature Database. The language of searched literature is limited to Chinese and English only, and the publication date is limited from creation to January 2023. Other ongoing and unpublished clinical trial registry studies will also be reviewed to obtain more relevant literature.

Disease qualifiers for the English literature search included “cerebral stroke,” “hemiplegia,” “sequela of apoplexy “, “dyskinesia”; intervention qualifiers included “electroacupuncture,” “acupuncture “, “acupunct,” “pharmacupuncture,” “auricular,” “fire needling,” “mirror therapy”; the study type word was limited to “randomized controlled trial”. Using Pubmed as an example, the detailed search strategy is shown in Table 1 below.

**Table 1:** PubMed search strategy.

No.	Search items
#1	hemiplegia [MeSH]
#2	Monoplegia [Title/Abstract]
#3	Hemiplegias [Title/Abstract]
#4	Hemiplegia, Post-Ictal [Title/Abstract]
#5	Crossed Hemiplegia [Title/Abstract]
#6	Spastic Hemiplegia [Title/Abstract]
#7	dyskinesia [Title/Abstract]
#8	#1 OR #2-7
#9	Acupuncture [MeSH]
#10	Electroacupuncture [Title/Abstract]
#11	Dermal needle [Title/Abstract]
#12	Acupuncture Therapy [Title/Abstract]
#13	Pharmacoacupuncture Treatment [Title/Abstract]
#14	Acupotomies [Title/Abstract]
#15	#9 OR #10-14
#16	Mirror Movement Therapy [Title/Abstract]
#17	Mirror Movement Therapies [Title/Abstract]
#18	Mirror Therapy [Title/Abstract]
#19	mirror box [Title/Abstract]
#20	visual mirror feedback [Title/Abstract]
#21	#16 OR #17-20
#22	Randomized controlled trial (all field)
#23	Controlled clinical trial (all field)
#24	Randomized (all field)
#25	Random allocation (all field)
#26	Randomly (all field)
#27	Placebo (all field)
#28	Double-blind method (all field)
#29	Single-blind method (all field)
#30	Trials (all field)
#31	#22 OR #23-30
#32	#8 AND #15 AND #21 AND #31

### 2.3. Inclusion criteria

This study included only randomized controlled clinical trials (RCTs) of AT combined with MT for post-stroke dyskinesia. Studies will be excluded if they are conference papers, editorials, case reports, and crossover studies. All patients should be diagnosed with post-stroke dyskinesia with reference to the Chinese Guidelines for the Prevention and Treatment of Cerebrovascular Diseases,^[16]^ the Chinese Guidelines and Consensus on the Diagnosis and Treatment of Cerebrovascular Diseases,^[17]^ the Diagnostic Efficacy Criteria for Chinese Medical Evidence,^[18]^ the Guidelines for Clinical Research on New Chinese Medicines^[19]^ and the patients included in this study had a confirmed stroke on head computed tomography or magnetic resonance imaging. All patients included in this study were hospitalized patients. All study participants were hospitalized, with the duration of their condition spanning from 2 weeks to 1 year, covering subacute, recovery, and sequelae phases of stroke. There were no restrictions based on age, gender, or race.

### 2.4. Exclusion criteria

Clinical trial subjects with cognitive or comprehension impairments unable to cooperate in completing the trial; studies for which abstracts or full texts are not available or that lacking important clinical data; duplicate publications with only 1 taken; exclusion of reviews, mechanistic discussions, and animal testing literature; those with obvious errors and loopholes in the trial design.

### 2.5. Intervention types

The experimental group used AT combined with MT, and the control group used MT, AT, or RT. The forms of AT included milli-needle, electro-acupuncture, head acupuncture, fire acupuncture, etc, and the selection of acupuncture points, acupuncture technique, and treatment time was not limited.

### 2.6. Outcome measurements

Total effective rate, Fugl–Meyer assessment scale (FMA) upper and lower limb scores, modified Barthel index (MBI) score, Berg balance scale (BBS) score, and modified Ashworth scale (MAS) score were used as outcome indicators to evaluate the improvement of motor function in patients with AT combined with MT.

### 2.7. Data extraction and assessment of risk of bias

Data were extracted independently by 2 researchers (PYF, HSQ) using a predetermined standard Excel spreadsheet for the final included studies. The main extracted contents were title, author, time of publication, intervention, acupuncture points, duration of treatment, sample size, patient gender, age, total effective rate, FMA upper and lower limb scores, MBI score, BBS score, and MAS score. When important information is missing from the studies, the authors can be contacted for details by email or telephone.

The quality of the included trials was assessed using the Cochrane Risk of Bias Assessment Tool^[20]^ by 2 reviewers (PYF and HSQ). The risk of bias was assessed from the following 7 domains: random sequence generation; allocation sequence concealment; blinding of participants and staff; blinding of outcome assessment; incomplete outcome data; selective reporting; and other biases. Assessments were categorized as low risk, high risk, and uncertain risk, and any disagreements between 2 investigators were resolved by negotiation with a third investigator.

### 2.8. Grading of Recommendations Assessment Development and Evaluation (GRADE) assessment

GRADE grading classifies the quality of evidence into 4 grades: high, medium, low, and very low.^[21]^ Considering that all the studies included in this study were RCTs, the quality of evidence grade was assessed comprehensively based on 5 downgrading factors. The 5 downgrading factors included risk of bias, inconsistency, indirectness, imprecision, and publication bias.

### 2.9. Synthesis and evaluation of data

The software Review Manager V.5.4, (The Cochrane collaboration) was used to carry out the meta-analysis. Continuous data was presented as the standardized mean difference (MD) with a 95% confidence interval (CI), whereas dichotomous data was reported as the risk ratio with a 95% CI. The significance level was set at 50% for the Higgins *I*^2^ test, which was used to examine heterogeneity. For the goal of meta-analysis, a model with fixed effects was employed if heterogeneity was not considerable (*I*^2^ ≤ 50%). If heterogeneity was significant (*I*^2^ ≥ 50%), a random-effects model was used. We performed sensitivity analysis and subgroup analysis to find their potential explanations.

## 3. Results

### 3.1. Study characteristics

**Figure 1.**
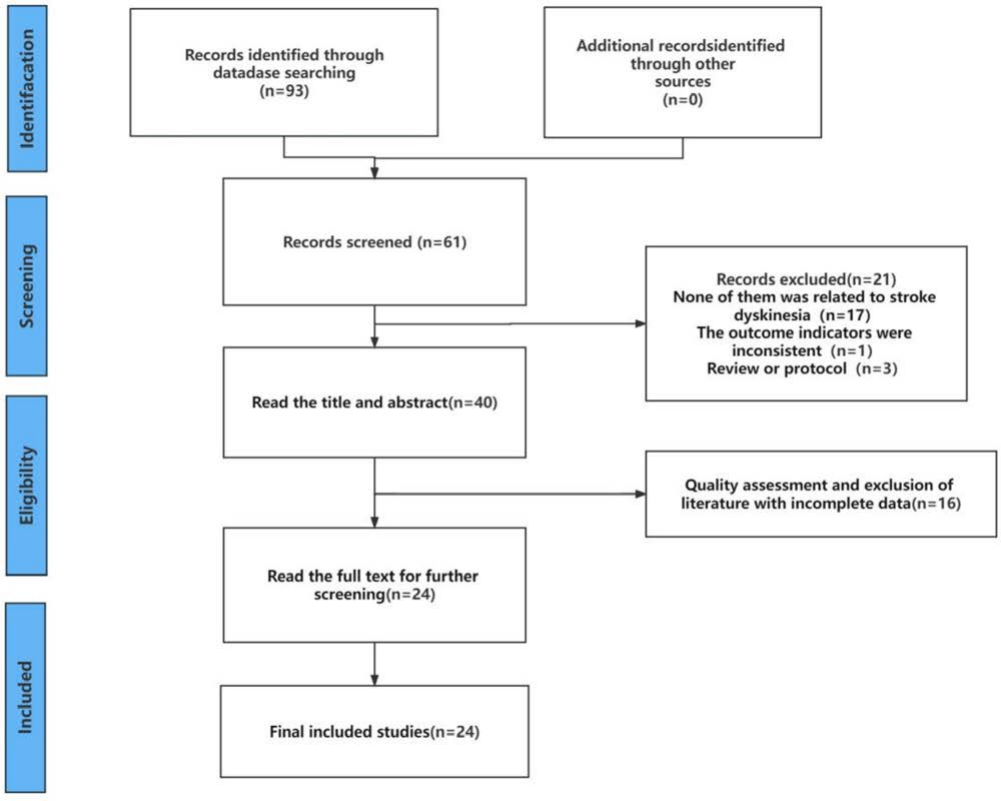
Literature screening process.

According to the preplanned search strategy and literature collection method, a total of 93 studies were retrieved from Chinese and English databases, and additional searches in the clinical research registry platform did not find studies on relevant topics. 32 studies were found by NoteExpress software check, and 61 studies remained after deletion. The titles and abstracts of the remaining studies were then read according to the inclusion and exclusion criteria, and 40 studies remained after screening. After reading the screened studies, there were finally 24 RCTs that met the criteria, and the detailed screening process of the literature is detailed in Figure 1.

The 24 included studies were all clinical RCTs^[22–45]^ with a total of 2133 patients. The minimum sample size of the trials was 40 cases and the maximum sample size was 120 cases, with patients ranging in age from 40 to 72 years. On the research objects, 14 studies included patients with upper limb movement disorders,^[22–33,44,45]^ 9 included patients with lower limb movement disorders,^[34–41,43]^ and 1 included patients with upper and lower limb movement disorders.^[42]^ In terms of interventions, all trial groups used AT + MT; 15 studies in the control group used MT,^[23,24,26–28,30,31,34,35,37–39,41–45]^ 7 studies used AT,^[23,24,32–34,40,43]^ 3 studies used conventional rehabilitation,^[22,25,27]^ and 2 studies used sham-AT + MT.^[29,36]^ In terms of outcome indicators, all 24 studies used the Fugl–Meyer Motor Function Scale, 15 studies used the MBI Livability Index,^[23,24,26,27,29–33,35,37–40,45]^ 7 studies used the BBS score,^[35–38,41,42]^ 5 studies used the MAS score,^[23,26,29,37,41]^ and 6 studies used the total effective rate.^[22,27,28,31,35,44]^ Details can be found in Table 2.

**Table 2:** Characteristics of analyzed trials.

Authors	Age			Sample size	Methods of intervention			Design	Main outcomes
	T	C1	C2		T	C1	C2		
Yang WD 2019^[22]^	62 ± 12	–	–	60	AT + MT	RT	–	2-arm	*^,^†
Yin ZL 2020^[23]^	55 ± 10	52 ± 11	53 ± 13	78	AT + MT	MT	AT	2-arm	†^,^§^,^¶
Zhang R 2017^[24]^	55.2 ± 10.9	54.9 ± 11	54.8 ± 10.1	60	AT + MT	MT	AT	2-arm	†
Li JM 2022^[25]^	68.35 ± 4.16	67.56 ± 3.77	–	94	AT + MT	RT	–	2-arm	†
Ma ZY 2019^[26]^	67.2 ± 5.7	65.4 ± 4.8	–	40	AT + MT	MT	–	2-arm	†^,^¶
Wang XT 2021^[27]^	53.97 ± 8.89	56.60 ± 9.44	54.80 ± 5.90	90	AT + MT	MT	RT	2-arm	*^,^†^,^§
Zhang YN 2020^[28]^	59.88 ± 6.6	58.51 ± 6.1	–	86	AT + MT	MT	–	2-arm	*^,^†^,^§
Duan C 2020^[29]^	58 ± 10	60 ± 11	–	100	AT + MT	Sham AT + MT	–	2-arm	†^,^§^,^¶^,^#
Zhang YN 2021^[30]^	51.93 ± 6.57	52.07 ± 6.62	–	86	AT + MT	MT	–	2-arm	†^,^¶
Xie JJ 2018^[31]^	56 ± 8	54 ± 6	–	90	AT + MT	MT	–	2-arm	*^,^†^,^§
Zhang N 2021^[32]^	58.81 ± 10.94	–	56.37 ± 9.53	64	AT + MT	–	AT	2-arm	†^,^§
Si LG 2021^[33]^	57.57 ± 10.59	–	61.06 ± 11.25	70	AT + MT	–	AT	2-arm	†^,^§
Cao KY 2022^[34]^	64.07 ± 10.25	63.51 ± 10.40	62.37 ± 11.49	81	AT + MT	MT	AT	Double lower limbs	‡
Chen LX 2022^[35]^	64.2 ± 8.8	63.8 ± 7.8	–	100	AT + MT	MT	–	Double lower limbs	*^,^‡^,^§^,^‖^,^¶
Wang AJ 2022^[36]^	62.20 ± 9.17	63.75 ± 8.56	–	108	AT + MT	Sham AT + MT	–	Double lower limbs	‡^,^‖
Ge C 2021^[37]^	55 ± 11	54 ± 12	–	75	AT + MT	MT	–	Double lower limbs	‡^,^§^,^‖
Chen LX 2021^[38]^	56 ± 12	58 ± 11	–	84	AT + MT	MT	–	Double lower limbs	‡^,^§^,^‖
Cui SY 2017^[39]^	51 ± 4	53 ± 4	–	65	AT + MT	MT	–	Double lower limbs	‡^,^§
Zhang XH 2018^[40]^	61.79 ± 2.07	–	61.85 ± 2.35	120	AT + MT	–	AT	Double lower limbs	‡^,^§
Yang Q 2020^[41]^	60 ± 6.98	59 ± 7.23	–	60	AT + MT	MT	–	Double lower limbs	‡^,^‖
Luo Q 2020^[42]^	63.28 ± 3.23	63.04 ± 3.16	–	96	AT + MT	MT	–	Bilateral upper and lower limbs	†^,^‡^,^‖
Zhu D 2019^[43]^	49 ± 3.7	54 ± 1.9	52 ± 2.3	120	AT + MT	MT	AT	Bilateral upper and lower limbs	‡^,^‖
Wang Y 2022^[44]^	65.84 ± 9.38	65.28 ± 9.93	–	96	AT + MT	MT	–	Bilateral upper and lower limbs	*^,^†
Wu JS 2022^[45]^	62.20 ± 5.14	62.35 ± 5.10	–	90	AT + MT	MT	–	Bilateral upper and lower limbs	†^,^§

AT = acupuncture therapy, C = control group, MT = mirror therapy, RT = rehabilitation therapy, T = test group.

*Total effective rate. †Fugl–Meyer assessment scale upper limb score. ‡Fugl–Meyer assessment scale lower limb score. §Modified Barthel index score. ‖Berg balance scale score. ¶Berg balance scale rating. #Adverse reaction.

Since MT was largely the same, only the acupuncture characteristics were included in this study. The types of acupuncture points used in the studies varied: 10 studies used scalp acupoints,^[23–27,35–38,43]^ 14 studies used body acupoints,^[22,29,33,34,39–42,44,45]^ and 3 studies used both scalp and body acupoints.^[28,31,32]^ the deqi sensation was reported in 10 studies,^[24,26,28–30,34,39,40,42,45]^ twirling in 15 studies,^[23–27,31–33,35–38]^ electric stimulation in 2 studies,^[22,41]^ and warm acupuncture in 1 study.^[42]^ 14 studies^[23,24,26–31,37,39–42,45]^ conducted treatments over a period of 4 weeks, 3 studies^[25,32,34]^ for 6 weeks, 5 studies^[23,35,36,38,43]^ for 8 weeks, 1 study^[33]^ for 2 weeks and 1 study^[44]^ for 12 weeks. Details can be found in Table 3.

**Table 3:** Acupuncture characteristics of the included studies.

Authors	Type of acupuncture	Stimulation response	Acupoints	Intervention frequency	Treatment duration
Yang WD 2019^[22]^	ES + BA	1~4 mA, 2/15 Hz	SJ14, LI15, LI5, LI4, SJ5, LI11	5 times a wk 30 min each time	4 wk
Yin ZL 2020^[23]^	SA	Twirling	Upper 1/5 and middle 2/5 of the parietal temporal anterior oblique line and upper 1/5 and middle 2/5 of the parietal temporal posterior oblique line	5 times a wk 40 min each time	8 wk
Zhang R 2017^[24]^	SA	Deqi sensation, twirling	DU20, EX-HN1, lateral parietal temporal anterior oblique line	6 times a wk 20 min each time	4 wk
Li JM 2022^[25]^	SA	Twirling	DU21, SI16, BL7, BL6, GB17, GB16, DU20, EX-HN1	5 times a wk 6 h each time	6 wk
Ma ZY 2019^[26]^	SA	Deqi sensation, twirling	Parietal midline, anterior parietal temporal oblique line, and posterior parietal temporal oblique line	6 times a wk 25 min each time	4 wk
Wang XT 2021^[27]^	SA	Twirling	Anterior parietal temporal oblique line and posterior parietal temporal oblique line	5 times a wk 40 min each time	4 wk
Zhang YN 2020^[28]^	SA + BA	Deqi sensation	DU20, EX-HN1, LI11, PC6, LI4, ST36, SP6, LR3	5 times a wk 30 min each time	4 wk
Duan C 2020^[29]^	BA	Deqi sensation	LR3, KI3, DU26, PC6, SP6, HT1, LU5, ST36, LI15, LI4	5 times a wk 20 min each time	4 wk
Zhang YN 2021^[30]^	BA	Deqi sensation	EX-UE9	6 times a wk 30 min each time	4 wk
Xie JJ 2018^[31]^	SA + BA	Twirling	Anterior parietal temporal oblique line, SJ13, SJ11, LI15, LI11, LI10, LI5, LI4	5 times a wk 30 min each time	4 wk
Zhang N 2021^[32]^	SA + BA	Twirling	Jin 3 needles	6 times a wk 30 min each time	6 wk
Si LG 2021^[33]^	BA	Twirling	LI15, SJ14, LI14, LI10, LI11, LU5, LI4, SJ5, SI4, EX-UE9	5 times a wk 30 min each time	2 wk
Cao KY 2022^[34]^	BA	Deqi sensation	ST36, ST-40, GB39, LR3, KI3, GB40, KI6	5 times a wk 20 min each time	6 wk
Chen LX 2022^[35]^	SA	Twirling	DU20, EX-HN5, anteroposterior oblique line of the parietal temporal	5 times a wk 30 min each time	8 wk
Wang AJ 2022^[36]^	SA	Twirling	Anteroposterior oblique line of the parietal temporal	5 times a wk 40 min each time	8 wk
Ge C 2021^[37]^	SA	Twirling	Anteroposterior oblique line of the parietal temporal	5 times a wk 40 min each time	4 wk
Chen LX 2021^[38]^	SA	Twirling	Anteroposterior oblique line of the parietal temporal	5 times a wk 60 min each time	8 wk
Cui SY 2017^[39]^	BA	Deqi sensation	Jin 3 needles	6 times a wk 30 min each time	4 wk
Zhang XH 2018^[40]^	BA	Deqi sensation	Jin 3 needles	5 times a wk 30 min each time	4 wk
Yang Q 2020^[41]^	ES + BA	10 mA, 100 Hz	ST31, GB28, BL36, LR9, GB31, BL37, ST36, GB34, GB39	5 times a wk 30 min each time	4 wk
Luo Q 2020^[42]^	BA	Deqi sensation, warm AT	EX-B2, PC2, LU5, EX-UE, LI4, BL37, ST32, ST34, SP9, SP6	5 times a wk Time not specified	4 wk
Zhu D 2019^[43]^	SA	Twirling	Anteroposterior oblique line of the parietal temporal	3 times a wk 60 min each time	8 wk
Wang Y 2022^[44]^	BA	Twirling	SP6, DU26, HT1, LU5, BL40, PC9, KI1	five times a wk 30 min each time	12 wk
Wu JS 2022^[45]^	BA	Deqi sensation, twirling	LI11, LI15, LI4, SJ5, SJ3, SI3	6 times a wk 20 min each time	4 wk

AT = acupuncture therapy, BA = body acupuncture, C = control group, ES = electrical stimulation, SA = scalp acupuncture, T = test group.

### 3.2. Quality assessment

**Figure 2.**
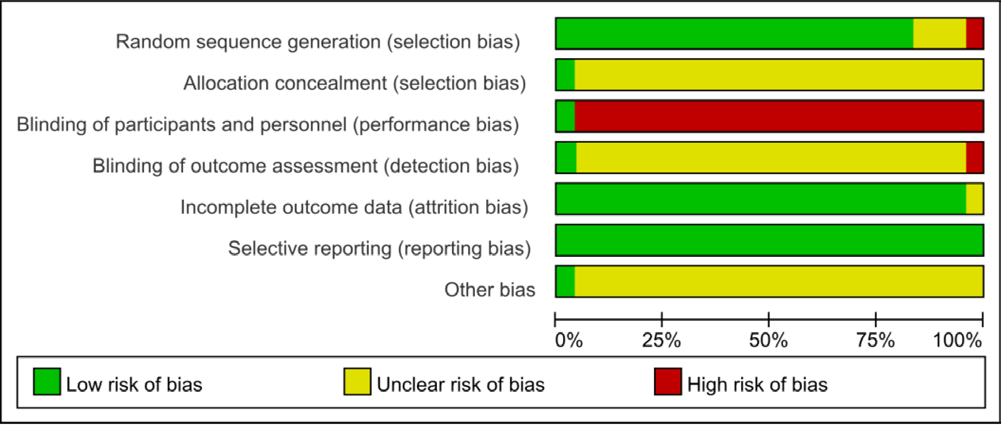
The proportion of articles included in the quality assessment of acupuncture combined with mirror therapy for post-stroke dyskinesia.

**Figure 3.**
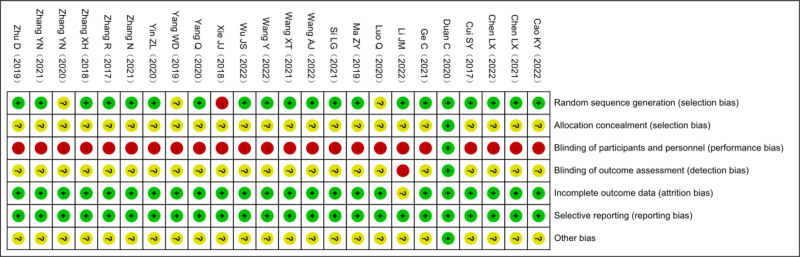
Acupuncture combined with mirror therapy for post-stroke dyskinesia was included in the quality evaluation of the literature.

All 24 included studies mentioned random grouping in the text; 20 reported the appropriate method of random sequence generation and all used the random number table method^[23–27,29,30,32–41,43–45]^; 1 was randomly grouped according to the order of enrollment,^[31]^ with a high risk of bias; 3 studies did not mention the method of random assignment^[22,28,42]^ and only mentioned the word “random,” so the risk of bias was uncertain. In aspect of allocation concealment and blinded implementation, only 1 study reported the allocation concealment scheme in detail, and the remaining 23 studies did not report the method of allocation concealment.^[29]^ As for blinded implementation, only 1 study implemented blinded methods for the investigators, operators, and data raters,^[29]^ and none of the remaining studies reported whether blinded methods were used, as detailed in Figures 2 and 3.

### 3.3. Total efficiency

**Figure 4.**
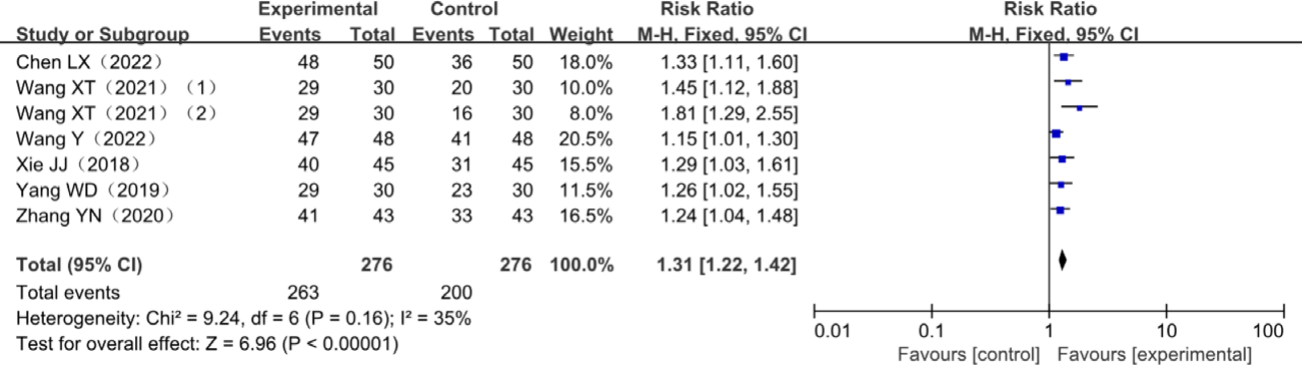
Meta-analysis forest plot of total efficiency.

A total of 6 trials reported the clinical efficacy of AT combined with MT in stroke patients, among them 1 three-arm trial^[27]^ involving a total of 522 patients with post-stroke dyskinesia, including 246 cases in the trial group and 276 cases in the control group. Heterogeneity test *χ*^2^ = 9.24, df = 6, *P* = .16, *I*^2^ = 35%, indicating homogeneity among the included studies, making it suitable for meta-analysis with fixed effects model; meanwhile, the combined effect size (relative risk = 1.31, 95% CI [1.22–1.42], *Z* = 6.96, *P* < .0001) showed the difference between the 2 groups was statistically significant. The analysis results are detailed in Figure 4.

### 3.4. FMA upper limb score

**Figure 5.**
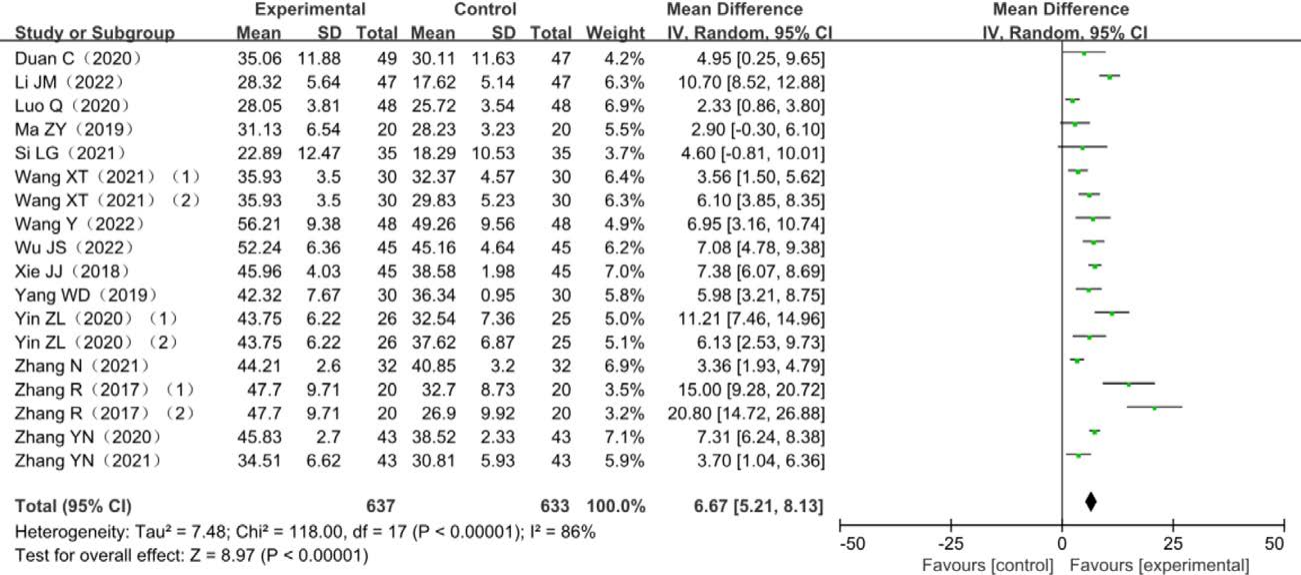
Meta-analysis forest plot of the FMA upper limb score. FMA = Fugl–Meyer assessment scale.

A total of 15 studies reported changes in FMA upper limb scale scores, including 3 three-arm trials,^[23,24,27]^ a total of 1194 patients with post-stroke dyskinesia included, among which 561 patients in the trial group and 633 in the control group. The heterogeneity test *χ*^2^ = 118.00, df = 17, *P* < .00001, and *I*^2^ = 86% suggested significant heterogeneity among studies and it was suitable for meta-analysis with a random-effects model. Fifteen studies combined effect sizes (MD = 6.67, 95% CI [5.21–8.13], *Z* = 8.97, *P* < .00001) showed the differences were statistically significant. The results of the analysis are detailed in Figure 5.

### 3.5. FMA lower limb score

**Figure 6.**
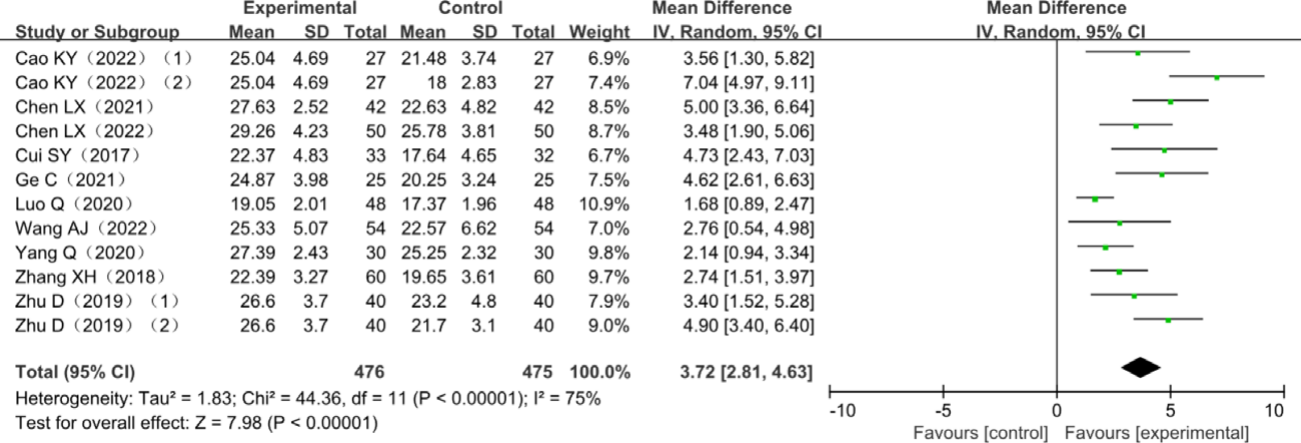
Forest plot of meta-analysis of FMA lower limb scores. FMA = Fugl–Meyer assessment scale.

A total of 10 studies reported changes in FMA lower limb scores, including 2 three-arm trials,^[34,43]^ a total of 884 patients with lower limb dyskinesia after stroke included, among which 409 patients in the trial group and 475 in the control group. The heterogeneity test *χ*^2^ = 44.36, df = 11, *P* = .006, *I*^2^ = 75%, suggested significant heterogeneity among studies and it was suitable for meta-analysis with a random-effects model. Ten studies combined effect size (MD = 3.72, 95% CI [2.81–4.63], *Z* = 7.98, *P* < .00001) showed the differences were statistically significant. The results of the analysis are detailed in Figure 6.

### 3.6. MBI score

**Figure 7.**
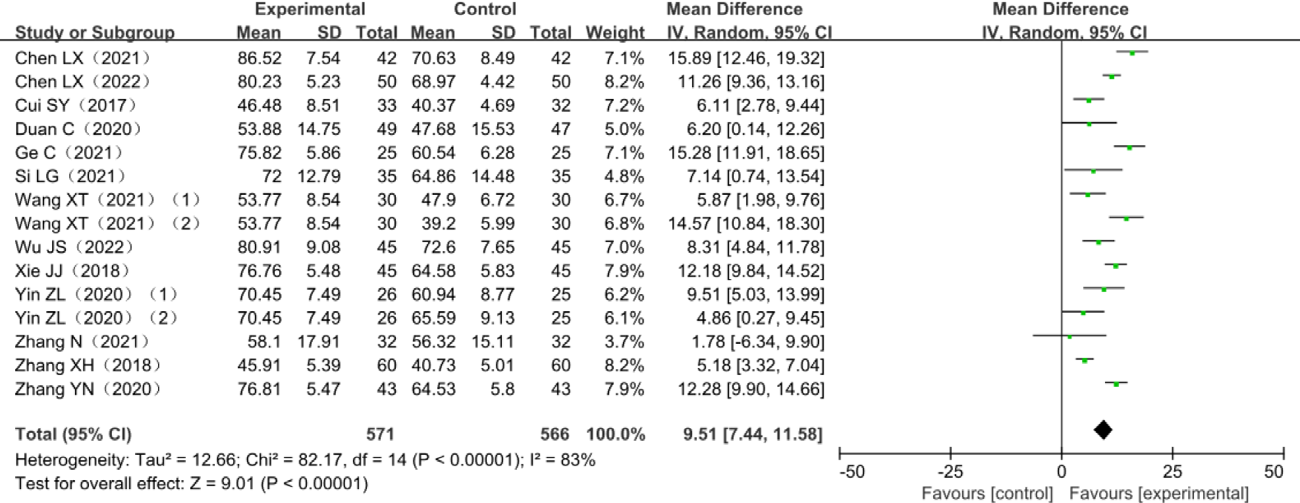
Meta-analysis forest plot of MBI scores. MBI = modified Barthel index.

A total of 13 studies reported changes in MBI scores after treatment with AT + MT, and 1 was a 3-arm trial,^[23,27]^ 1081 patients with post-stroke dyskinesia included, among which 515 in the trial group and 566 in the control group. The heterogeneity test *χ*^2^ = 82.17, df = 14, *P* < .00001, *I*^2^ = 83%, suggested significant heterogeneity among studies and it was suitable for meta-analysis with a random-effects model. Thirteen studies combined effect sizes (MD = 9.51, 95% CI [7.44–11.58], *Z* = 9.01, *P* < .00001) showed the differences were statistically significant. The results of the analysis are detailed in Figure 7.

### 3.7. BBS score

**Figure 8.**
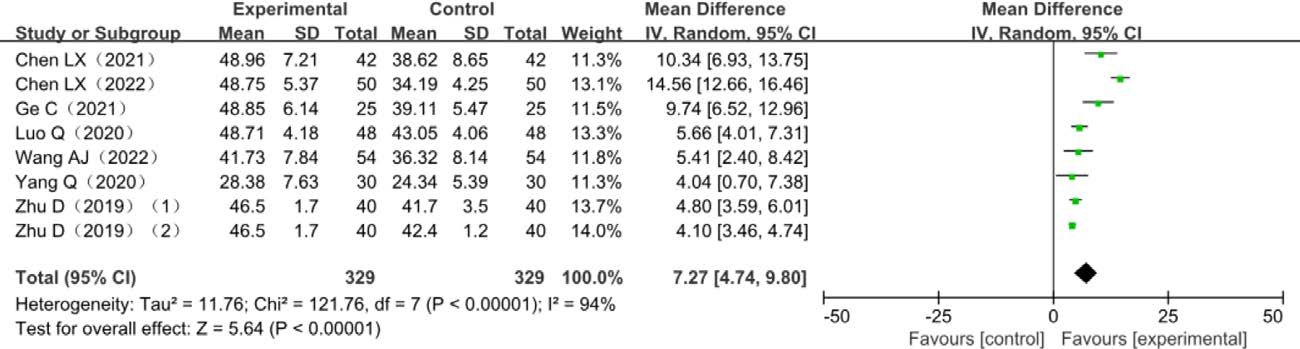
Meta-analysis forest plot for BBS score comparison. BBS = Berg balance scale.

Seven studies reported on changes in BBS scores, and 1 was a 3-arm trial^[43]^ that included 618 patients with post-stroke dyskinesia, among which 289 in the trial group and 329 in the control group. The control group was all on MT. The heterogeneity test *χ*^2^ = 121.76, df = 7, *P* < .00001, *I*^2^ = 94%, suggested significant heterogeneity among studies and it was suitable for meta-analysis with random effects model. The combined effect size (MD = 7.27.94, 95% CI [4.74–9.80], *Z* = 4.85, *P* < .00001) showed the difference was statistically significant. The results of the analysis are detailed in Figure 8.

### 3.8. MAS score

**Figure 9.**
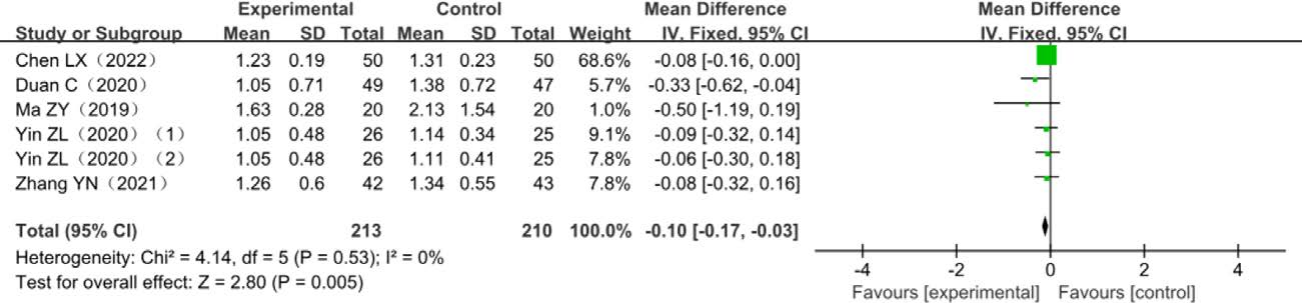
Forest plot of meta-analysis of MAS scores. MAS = modified Ashworth scale.

Five studies reported changes in MAS scores, and 1 was a 3-arm trial,^[23]^ which included 397 patients with post-stroke dyskinesia, among which 187 were in the trial group and 210 in the control group. The heterogeneity test *χ*^2^ = 4.14, df = 5, *P* = .53, *I*^2^ = 0%, indicated homogeneity between studies and it was suitable for meta-analysis with a fixed-effects model. The combined effect size (MD = −0.10, 95% CI [−0.17–−0.03], *Z* = 2.80, *P* = .005) showed the difference was statistically significant. The results of the analysis are detailed in Figure 9.

### 3.9. Adverse reactions

One study^[29]^ reported the occurrence of adverse reactions, mainly including pain and bleeding at the acupuncture site, which did not affect the subsequent treatment. One study^[29,34]^ reported no serious adverse reactions, while the remaining studies did not report whether there were related adverse reactions, so the safety of AT combined with MT remains to be clarified.

### 3.10. Subgroup analysis

#### 3.10.1. FMA upper limb score subgroup analysis

**Figure 10.**
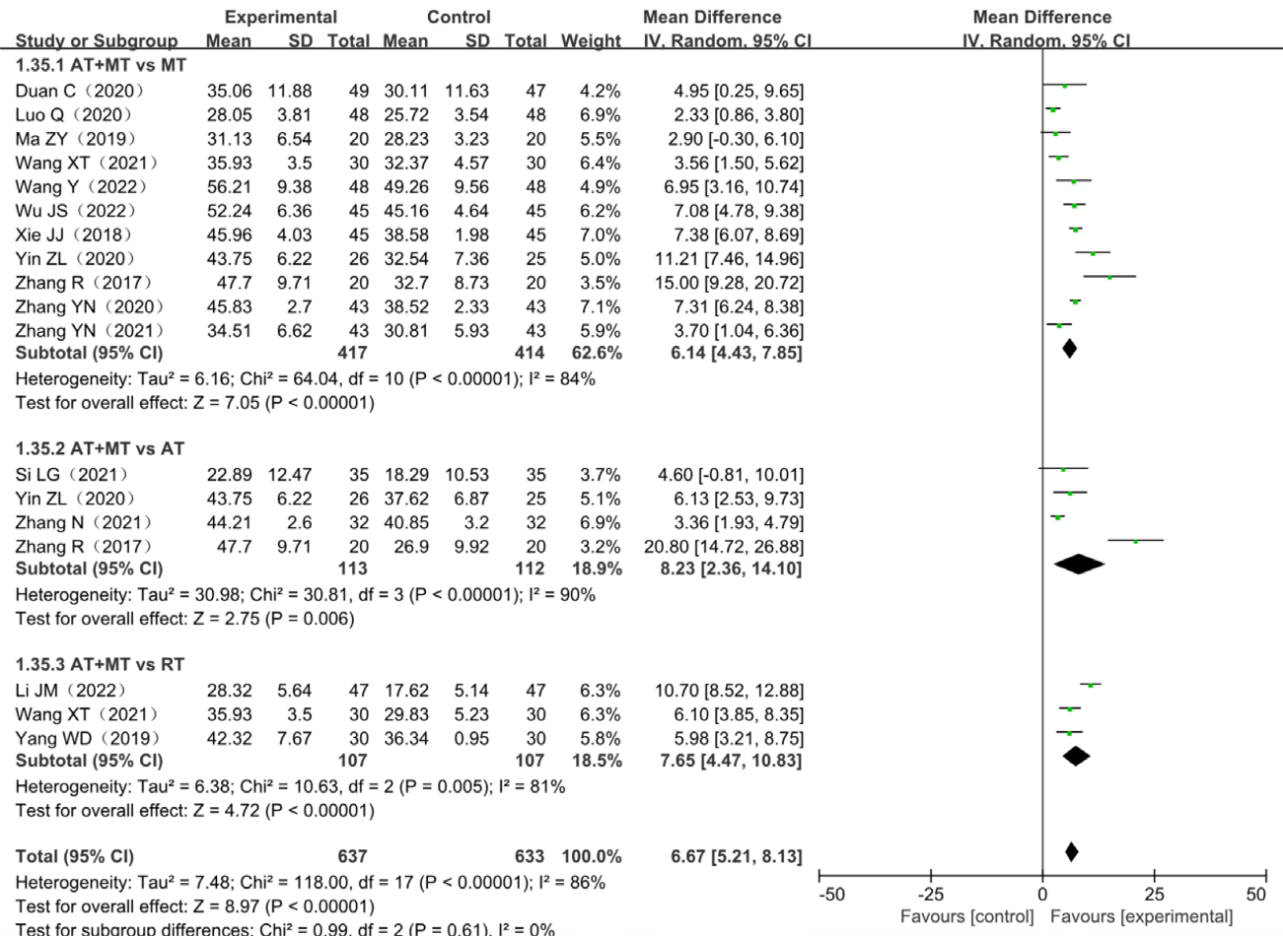
Forest plot for subgroup analysis of FMA upper limb scores. FMA = Fugl–Meyer assessment scale.

Considering the high heterogeneity among the studies, the 15 studies were grouped according to the different treatment methods of the control group, which was divided into MT group, AT group, and RT group. Compared with the monotherapy group, the FMA upper limb score of the combined treatment group was statistically increased in all 3 subgroups. (Compared with MT group: MD = 6.23, 95% CI [4.44–8.02], *Z* = 6.82, *P* < .00001; Compared with AT group: MD = 8.23, 95% CI [2.36–14.10], *P* < .05; Compared with RT group: MD = 7.65, 95% CI [4.47–10.83], *P* < .0001). The results of the analysis are detailed in Figure 10.

#### 3.10.2. FMA lower limb score subgroup analysis

**Figure 11.**
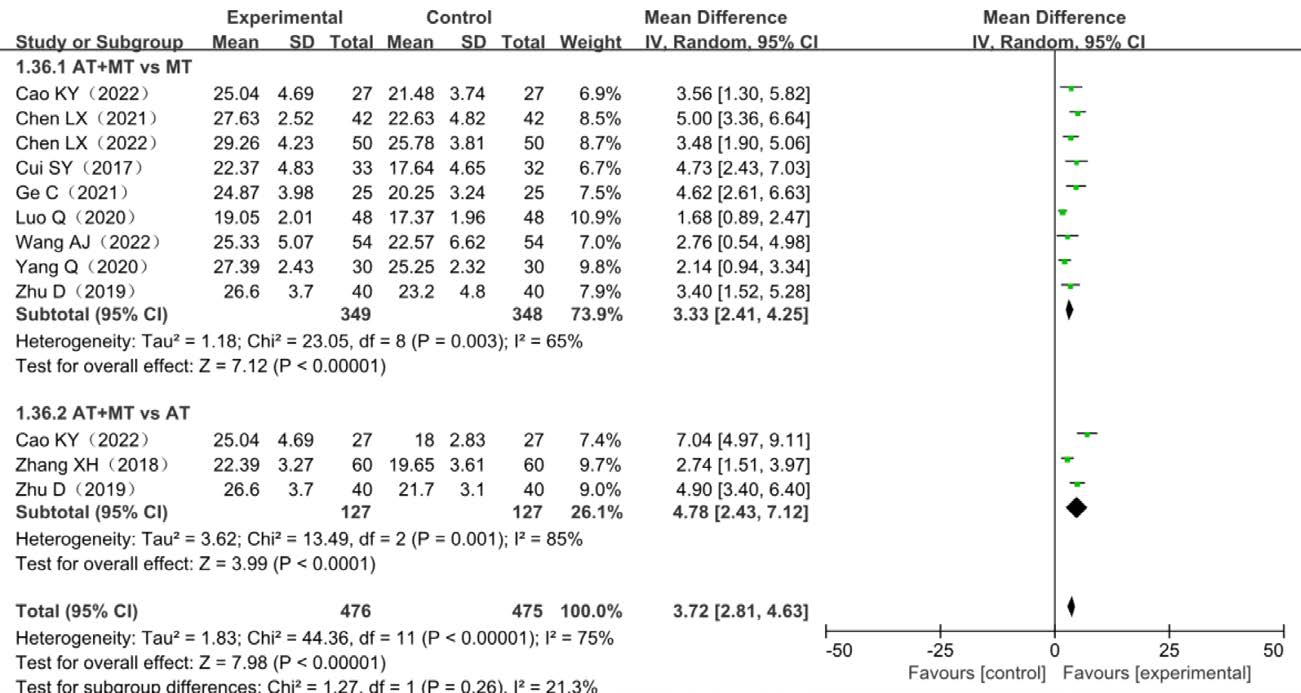
Forest plot of FMA lower limb score subgroup analysis. FMA = Fugl–Meyer assessment scale.

Considering the high heterogeneity among the studies, the 10 studies were grouped according to the different treatment methods of the control group, which was divided into the MT group and the AT group. Compared with the monotherapy group, the FMA upper limb score in the combined treatment group was statistically increased in all 2 subgroups. (Compared with MT group: MD = 3.33, 95% CI [2.41–4.25], *Z* = 7.12, *P* < .0001; Compared with AT group: MD = 4.78, 95% CI [2.43–7.12], *Z* = 3.99, *P* < .0001). The results of the analysis are detailed in Figure 11.

### 3.11. Sensitivity analysis

According to the sensitivity analysis of exclusion method, taking intervention measures as the condition of subgroup analysis, in the sensitivity analysis of FMA upper limb score, *I*^2^ = 3% between AT + MT and AT subgroup was found after excluding Zhang R (2017); meanwhile, the heterogeneity was greatly reduced, the *P* value of the result was still <0.05, and the trend of the result did not change. It can be considered that Zhang R (2017) is the source of heterogeneity in this analysis. *I*^2^ = 0% between AT + MT and RT was found after removing Li JM (2022); meanwhile. the heterogeneity was greatly reduced, the *P* value of the result was still <0.05, and the trend of the result did not change, which could be considered that Li JM (2022) was the source of heterogeneity in this analysis. In the sensitivity analysis of FMA lower limb score, *I*^2^ = 43% was found after excluding Luo Q (2020), and the heterogeneity was greatly reduced between AT + MT and AT subgroup, the *P* value of the result was still <0.05, and the trend of the result did not change, which could be considered that Luo Q (2020) was the source of heterogeneity in this analysis. The sensitivity analysis of MBI score showed that *I*^2^ was stable after eliminating studies one by one.

### 3.12. Bias of publication

#### 3.12.1. Publication bias of the upper limb FMA score

**Figure 12.**
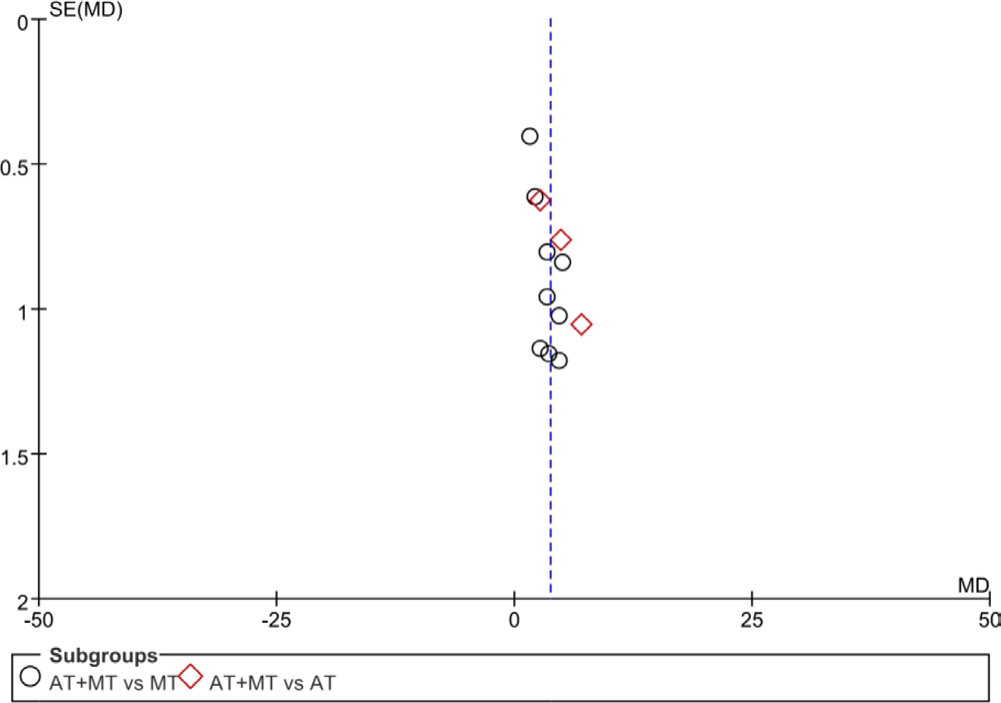
Funnel plot of FMA upper limb score. FMA = Fugl–Meyer assessment scale.

The funnel plot was drawn according to the FMA upper limb score, and the 95% CI was not marked with a dashed line in the plot because the study took a random-effects model to combine effect sizes. The scatter plot had a small discrete trend, mostly concentrated at the top and middle of the funnel plot, with good symmetry, suggesting that there was no reporting bias in the study. The analysis results are detailed in Figure 12.

#### 3.12.2. Publication bias of the lower limb FMA score

**Figure 13.**
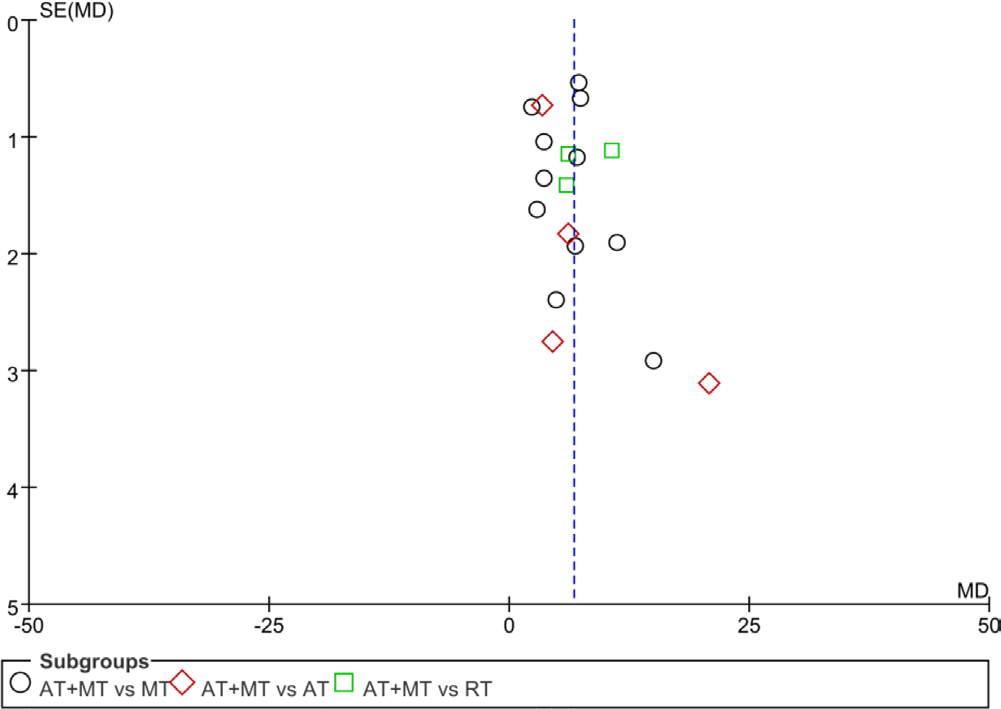
FAM lower limb score funnel plot.

The funnel plot was drawn according to the FMA upper limb score, and the 95% CI was not marked with a dashed line in the plot because the study took a random-effects model to combine the effect sizes. The scatter plot had a small discrete trend, mostly concentrated in the top and middle of the funnel plot, with poor symmetry and a missing corner in the lower left corner, suggesting the existence of a certain publication bias. The analysis results are detailed in Figure 13.

### 3.13. GRADE evidence quality assessment

The quality of GRADE evidence was evaluated by 6 outcome indicators of total effective rate, FAM upper limb score, FAM lower limb score, MBI score, BBS score, and MAS score, and the results showed that the MAS score was evaluated as intermediate, the total effective rate and lower limb FMA score were both evaluated as low, and the remaining indicators were evaluated as very low. It is suggested that the results of this study still need more high-quality RCT evidence-based support, and the analysis results are detailed in Table 4.

**Table 4:** GRADE evidence quality and strength of recommendation.

Indicators	Number of RCTs	Quality assessment					Effect value (95% CI)	Level of evidence
		Risk of bias	Inconsistency	Indirectness	Inaccuracy	Publication bias		
Total efficiency	6^[22,27,28,31,38,44]^	Serious*	None	None	None	Serious§	RR = 1.31, 95% CI (1.22–1.42)	Low
FMA upper limb score	15^[22–33,42,44,45]^	Serious*	Serious†	None	Serious‡	Serious§	MD = 6.67, 95% CI (5.21–8.13)	Extremely low
FMA lower limb score	10^[34–43]^	Serious*	Serious†	None	None	None	MD = 3.72, 95% CI (2.81–4.63)	Low
MBI score	13^[23,24,26,27,29–33,35,37–40,45]^	Serious*	Serious†	None	Serious‡	Serious§	MD = 9.51, 95% CI (7.44–11.58)	Extremely low
BBS score	7^[36–39,42,43]^	Serious*	Serious†	None	Serious‡	None	MD = 7.27, 95% CI (4.74–9.80)	Extremely low
MAS score	5^[23,26,29,37,41]^	Serious*	None	None	None	None	MD = −0.10, 95% CI (0.17–−0.03)	Middle

BBS = Berg balance scale, CI = confidence interval, FMA = Fugl–Meyer assessment scale, GRADE = Grading of Recommendations Assessment Development and Evaluation, MAS = modified Ashworth scale, MBI = modified Barthel index, MD = mean difference, RCT = randomized controlled trial, RR = relative risk.

*Blind and distribution concealment were not used. †Heterogeneity in sample size, interventions, and regimens between studies, *I*^2^ > 50%. ‡Small sample size and wide confidence interval. §Funnel plot suggests publication bias.

## 4. Discussion

### 4.1. Understanding of traditional Chinese medicine and modern medicine on post-stroke dyskinesia

The first detailed introduction to post-stroke dyskinesia came from Zhang Zhongjing of the Eastern Han Dynasty, and there was a special article on post-stroke dyskinesia. It is considered that “external wind” is the main cause of hemiplegia after stroke, and the severity of stroke is divided into 4 stages. Sun Simiao and Ge Hong used highly condensed objective signs as the diagnostic basis for post-stroke dyskinesia, such as irregular walking, limbs not retraction, tongue not turning, language astringent, etc. The judgment method was simple and feasible. In the Song, Jin, and Yuan Dynasties, with the development of traditional Chinese medicine, various Chinese doctors had a deeper understanding of the symptoms after stroke, and believed that the etiology and pathogenesis of stroke were mainly internal wind, such as Zhang Jingyue and Ye Tianshi. It makes the understanding of the etiology and pathogenesis of post-stroke dyskinesia more perfect.

The modern medical definition of stroke refers to the sudden rupture or blockage of blood vessels in the brain, resulting in hypoxic-ischemic damage to brain tissue. One of the most common sequelae after stroke is dyskinesia, which is 1% to 4% of all strokes^[46]^ and 22%^[47]^ of all secondary dyskinesia. There are many kinds of stroke secondary dyskinesia, which can be divided into hyperkinetic dyskinesia with excessive and abnormal involuntary movements and kinetic dyskinesia with bradykinesia. Dyskinesia is notably prevalent in cases involving injury to the middle cerebral artery, which is because the cortical branches of the middle cerebral artery supply nutrients in blood to areas of the lateral cerebral hemisphere, including those responsible for motor functions. Furthermore, the motor conduction system, which is essential for limb movement, comprises upper motor neurons, lower motor neurons, the extrapyramidal system, and the cerebellar system. Upper motor neurons play a critical role in sending voluntary movement signals to lower motor neurons. Damage to upper motor neurons can impair their ability to regulate muscle contraction, leading to central disorders such as spastic limb dyskinesia. On the other hand, lower motor neurons relay signals from the extrapyramidal system and cerebellar systems to muscles, inducing contraction through various nerve roots. When lower motor neurons are damaged, the function of triggering muscle contraction will be lost, leading to bradykinesia Hence, the location of a stroke within the brain determines the specific physical manifestations.

The recovery of neurological function in the early stage of stroke is mainly due to the pathological mechanism of brain plasticity and functional reorganization. This plasticity in the acute stage is manifested as vasoreflex contraction, reduction of edema in brain lesions, reduction of intracranial pressure, and hypometabolism of peripheral nerve cells in some necrotic areas due to the reduction of blood supply and energy metabolism. It plays a protective role in the brain by reducing oxygen consumption, energy consumption, lactic acid accumulation, metabolic acidosis, and improving the energy metabolism of brain cells. Therefore, early rehabilitation intervention of stroke may accelerate the plasticity and functional reorganization of the brain, and play an important role in the improvement of post-stroke dyskinesia.

### 4.2. Advantages of AT combined with MT in the treatment of post-stroke dyskinesia

Existing studies^[48,49]^ have demonstrated that there are changes in brain structure and neural connectivity after stroke, indicating some plasticity in the brain, which is a prerequisite for stroke recovery. Although many patients experience some degree of brain self-recovery, that is, some degree of improvement in physical function and activity,^[50]^ it is still incomplete. The intervention of external stimuli is still required. There is still a lack of effective clinical rehabilitation interventions for patients with stroke.^[51]^ As a relatively inexpensive and safe treatment, AT has been widely used to improve limb motor function, sensory function, swallowing function, and speech function after stroke. One study^[52]^ showed that AT promoted cell proliferation in the central nervous system of rats, regulated the blood flow velocity of the brain, promoted angiogenesis in brain lesion areas, regulated the differentiation and proliferation of neural stem cells, reshaped neurons, promoted nerve repair and regeneration, and activated specific motor areas of the cortex, thereby promoting neurological functional recovery. Clinical^[53,54]^ Studies and systematic reviews^[55,56]^ have also shown AT as a promising intervention that can improve motor and language function as well as activities of daily living.

MT has been widely used for motor recovery in stroke patients with hemiplegia. During the standardized MT procedure, patients saw the reflection of their healthy limb in a mirror located in the midsagittal plane, while the affected limbs were hidden behind the mirror so that the subject could not see it. This form of optical illusion or mirror visual feedback is based on the mirror neuron system, using the principle of planar mirror imaging, to project the contralateral active image onto the affected side. It also combines optical illusion, visual feedback, and virtual reality^[7]^ to stimulate the excitability of the affected cerebral hemisphere, equilibrate the excitation-inhibition balance between hemispheres, and promote the recovery of limb and hand function,^[11]^ which is simple, convenient, effective, and free of pharmacological side effects.^[57]^ The early rehabilitation intervention of MT can improve the limb dysfunction of patients with stroke more effectively.^[58]^

Based on the plasticity theory of the central nervous system, AT and MT can both stimulate nerve cells in different ways to remodel the nerve pathway. Therefore, AT combined with MT can accelerate the remodeling of cranial nerves and improve the therapeutic effect, and there have been good clinical reports.^[23,31,42]^

### 4.3. Analysis of quality evaluation results

The methodological quality of the 24 studies included in this study was generally low, among which 20 studies (83.33%) selected random number tables as the method of random sequence generation. In terms of the allocation concealment scheme and blinding method, only 1 study (4.17%) reported the allocation concealment scheme in detail, and the blinding method was used for researchers, operators, and data evaluators. The remaining 23 studies did not report the allocation concealment scheme or whether the blinding method was used. Therefore, in the future, scholars should pay attention to the implementation of allocation concealment schemes and blinding methods in the RCT research of AT combined with MT on post-stroke dyskinesia.

### 4.4. Meta-analysis results analysis

Amount of total effective rate of combined effect is (relative risk = 1.31, 95% CI [1.22–1.42], *Z* = 6.96, *P* < .0001); FMA upper limbs rating scale consolidation effect quantity is (MD = 6.67, 95% CI [5.21–8.13], *Z* = 8.97, *P* < .00001); FMA lower extremity scale score is (MD = 3.72, 95% CI [2.81–4.63], *Z* = 7.98, *P* < .00001); the combined effect of MBI scores is (MD = 9.51, 95% CI [7.44–11.58], *Z* = 9.01, *P* < .00001); BBS score combined effect quantity is (MD = 7.27.94, 95% CI [4.74–9.80], *Z* = 4.85, *P* < .00001); MAS score combined effect quantity is (MD = 0.10, 95% CI [0.17–0.03], *Z* = 2.80, *P* = .005). It is suggested that compared with other single therapies, AT combined with MT is more effective in improving FMA score, MBI score, BBS score, and MAS score in the treatment of post-stroke dyskinesia.

### 4.5. Adverse reactions

One study^[29]^ reported the occurrence of adverse reactions, mainly manifested as pain and bleeding at the acupuncture site, which did not affect the subsequent treatment. One study^[29,34]^ reported no serious adverse reactions, and the remaining studies did not report whether there were related adverse events, so the safety of AT combined with MT remains to be clarified.

### 4.6. GRADE assessment

The results of GRADE evidence quality evaluation showed that only MSA score was rated as intermediate evidence, and the downgrade factor was a serious risk of bias. The main manifestation was that allocation concealment scheme and blinding method were not used. In terms of total effective rate and FMA lower limb score, the evidence quality of the included studies was rated as low. Analysis of its factors showed that the risk of bias was serious, manifested in the absence of allocation concealment scheme and blinding method. In addition, the total effective rate was also more serious in publication bias, which may be related to less negative results reported in the included studies. The inconsistency of FMA lower limb score is also serious, which may be related to the obvious heterogeneity of sample size, intervention measures, and treatment courses among those studies. In terms of FMA upper limb score, MBI score, and BBS score, the evidence quality of the included studies was rated as extremely low. Risk of bias, inconsistency, and imprecision were the main degradation factors. Therefore, the results of the above 3 outcome measures have low confidence.

In conclusion, AT combined with MT can synergistically enhance the efficacy of patients with post-stroke dyskinesia and improve the evaluation of various dimensions. Therefore, AT combined with MT is a new technique in the field of stroke rehabilitation that deserves further study and exploration.

### 4.7. Limitations

There are still some shortcomings in this study. Firstly, the included studies are all in Chinese, which leads to language bias to some extent. Secondly, the methodological and reporting quality of the included studies was generally low, mainly in terms of allocation concealment scheme not being implemented and blinding method not being implemented, which is prone to selection and implementation bias. The indicators included in the studies were mostly scale scores, lacking objective indicators. Finally, some of the outcome indicators were highly heterogeneous, which may be related to the duration of treatment, frequency of treatment, and selection of acupuncture points.

Therefore, in response to the above deficiencies, future studies on retrieval should be comprehensive and standardized, involving multiple languages, especially covering relevant countries with extensive research on TCM. As for the included literature, in terms of clinical study design, trial bias should be strictly controlled, allocation concealment scheme should be implemented (using sealed, light-impermeable envelopes, or airtight containers), blinding method should be used (for subjects, interventionists, and data statistical analysts), and CONSORT guidelines should be followed; in terms of treatment protocols, efficacy criteria should be unified, operational details of AT combined with MT should be optimized, and the “core set of indicators” should be constructed, so as to provide a more effective reference value for future studies.

## 5. Conclusion

First, compared with single therapy, AT combined with MT is more advantageous in improving the degree of limb spasticity in patients with post-stroke dyskinesia, and the quality of GRADE evidence is rated as moderate. Therefore, the credibility of this conclusion is relatively ideal, and AT combined with MT has a high reference value for improving limb spasticity in patients with post-stroke dyskinesia.

Second, compared to monotherapy, it has certain advantages in improving body balance function and daily living activity function of patients with post-stroke dyskinesia, and the quality of GRADE evidence evaluation is relatively low. Therefore, the conclusion needs to be treated with caution and needs to be confirmed by more relevant RCT studies with large samples and high quality.

## Author contributions

**Investigation:** Xiaona Du.

**Methodology:** Guanghui Zhang.

**Supervision:** Jiao Ma.

**Writing – original draft:** Yufeng Peng.

**Writing – review & editing:** Nan Li, Guanghui Zhang, Shouqiang Huang.

## References

[1] Chinese multidisciplinary expert consensus on stroke disease monitoring. Chin Med J (Engl). 2021;101:317–26.

[2] Cramer SC Chopp M. Recovery recapitulates ontogeny. Trends Neurosci. 2000;23:265–71. 10838596 10.1016/s0166-2236(00)01562-9

[3] Su DY. Meta-analysis of selective serotonin reuptake inhibitors in the treatment of post-stroke dyskinesia. Chongqing, China: Chongqing Medical University; 2022.

[4] Ford B Peela S Roberts C. Secondary prevention of ischemic stroke: updated guidelines from AHA/ASA. Am Fam Physician. 2022;105:99–102. 35029929

[5] Morkisch N Thieme H Dohle C. How to perform mirror therapy after stroke? Evidence from a meta-analysis. Restor Neurol Neurosci. 2019;37:421–35. 31424422 10.3233/RNN-190935

[6] Thieme H Morkisch N Mehrholz J. Mirror therapy for improving motor function after stroke. Cochrane Database Syst Rev. 2018;7:CD008449. 29993119 10.1002/14651858.CD008449.pub3 PMC6513639

[7] Ramachandran VS Rogers-Ramachandran D Cobb S. Touching the phantom limb. Nature. 1995;377:489–90. 7566144 10.1038/377489a0

[8] Lisalde-Rodríguez ME Garcia-Fernández JA. [Mirror therapy in hemiplegic patient]. Rev Neurol. 2016;62:28–36. 26677779

[9] Liepert J. [Evidence-based methods in motor rehabilitation after stroke]. Fortschr Neurol Psychiatr. 2012;80:388–93. 22760510 10.1055/s-0031-1299490

[10] He Y Li K Chen Q Yin J Bai D. Repetitive transcranial magnetic stimulation on motor recovery for patients with stroke: a PRISMA compliant systematic review and meta-analysis. Am J Phys Med Rehabil. 2020;99:99–108. 31361620 10.1097/PHM.0000000000001277

[11] Madhoun HY Tan B Feng Y Zhou Y Zhou C Yu L. Task-based mirror therapy enhances the upper limb motor function in subacute stroke patients: a randomized control trial. Eur J Phys Rehabil Med. 2020;56:265–71. 32214062 10.23736/S1973-9087.20.06070-0

[12] Zhu ZY Li Y Li LF. To explore the effect of acupuncture combined with rehabilitation training on the recovery of motor function in patients with early hemiplegia after stroke. Shaanxi Tradit Chin Med. 2018;39:397–9.

[13] Wang J Tian L Zhang Z. Scalp-acupuncture for patients with hemiplegic paralysis of acute ischaemic stroke: a randomized controlled clinical trial. J Tradit Chin Med. 2020;40:845–54. 33000586 10.19852/j.cnki.jtcm.2020.05.015

[14] Fan AY Miller DW Bolash B. Acupuncture’s role in solving the opioid epidemic: evidence, cost-effectiveness, and care availability for acupuncture as a primary, non-pharmacologic method for pain relief and management–white paper 2017. J Integr Med. 2017;15:411–25. 29103410 10.1016/S2095-4964(17)60378-9

[15] Shamseer L. Preferred reporting items for systematic review and meta-analysis protocols (PRISMA-P) 2015: elaboration and explanation. BMJ. 2016;354:i4086. 27444514 10.1136/bmj.i4086

[16] Rao ML. Guidelines for prevention and treatment of cerebrovascular diseases in China (trial version). Beijing, China: Department of Disease Control, Ministry of Health, Chinese Society of Neurology; 2007.

[17] Chinese guidelines and consensus for the diagnosis and treatment of cerebrovascular diseases (2016 edition). BeiJing: People’s Medical Publishing House. 2016.

[18] Diagnostic efficacy criteria of TCM diseases and syndromes. Beijing, China; 1994.

[19] Zheng YY. Guiding principles for clinical research of new traditional Chinese medicine. Beijing, China; 2002.

[20] Higgins JP Altman DG Gøtzsche PC. The Cochrane Collaboration’s tool for assessing risk of bias in randomised trials. BMJ. 2011;343:d5928. 22008217 10.1136/bmj.d5928 PMC3196245

[21] Balshem H Helfand M Schünemann HJ. GRADE guidelines: 3. Rating the quality of evidence. J Clin Epidemiol. 2011;64:401–6. 21208779 10.1016/j.jclinepi.2010.07.015

[22] Yang WD Ma YJ. To observe the first-stage effect of electroacupuncture combined with mirror therapy on shoulder-hand syndrome after stroke. Shanghai J Acupunct Moxibustion. 2019;38:492–6.

[23] Yin ZL Meng ZX Ge C. Clinical observation of interactive scalp acupuncture combined with task-oriented mirror therapy in the treatment of hemiplegic upper limb dysfunction after ischemic stroke. Chin Acupunct. 2020;40:918–22. 10.13703/j.0255-2930.20190819-0001 32959583

[24] Zhang R. To explore the effect of mirror therapy combined with Tongdu Xingshen acupuncture on the recovery of upper limb function in patients with stroke. Shanghai, China: Nanchang University; 2021.

[25] Li JM Chen WR Dang H. To explore the effect of scalp acupuncture cluster combined with mirror therapy on upper limb muscle tension and event-related potential in elderly patients with acute cerebral infarction and hemiplegia. Chin J Gerontol. 2022;42:3641–5.

[26] Ma ZY Xu MF Yu XF. Clinical study of scalp acupuncture combined with mirror therapy in the treatment of upper limb spastic paralysis after stroke. New Tradit Chin Med. 2019;51:182–5.

[27] Wang XT Hu Y Zhang DN. Effect of scalp acupuncture combined with mirror therapy on the recovery of upper limb motor function in patients with hemiplegia after cerebral infarction. Guide Tradit Chin Med. 2021;27:93–96 + 111.

[28] Zhang YN Yan X Gao WN. To investigate the effect of acupuncture combined with mirror therapy on upper limb motor function in patients with early stroke. Abstract of the latest medical information in the world. World Latest Medicine Information. 2020;20:186–7.

[29] Duan C Li ZL Xia WG. Effect of acupuncture combined with mirror therapy on upper limb motor function after stroke. Nerve injury and functional reconstruction. Neural Injury and Functional Reconstruction. 2020;15:155–8.

[30] Zhang YN Zheng P Yan X. Clinical study of acupuncture at Ba xie point combined with mirror therapy on upper limb and hand function in patients with hemiplegia after stroke. Rehabil China. 2021;36:353–5.

[31] Xie JJ Ll JX Sun Q. To observe the effect of acupuncture combined with mirror therapy on upper limb dysfunction of hemiplegia after stroke. Shanghai J Acupunct Moxibustion. 2018;37:494–8.

[32] Zhang N Xi SY Zheng GW. To explore the effect of acupuncture combined with mirror visual feedback training on upper limb function and activity of daily living in stroke patients with hemiplegia. Shandong Tradit Chin Med J. 2021;40:157–61.

[33] Si LG. Effect of mirror therapy combined with acupuncture on upper limb motor dysfunction after stroke. Yunnan, China: Yunnan University of Traditional Chinese Medicine; 2021.

[34] Cao KY Xie WY Wang QX. To observe the effect of mirror therapy combined with acupuncture therapy on the ankle dorsiflexion ability of the affected side in patients with stroke. Massage Rehabil Med. 2022;13:13–6.

[35] Chen LX Li CJ Yang AR. To investigate the effect of scalp acupuncture combined with mirror therapy on lower limb dysfunction and activity of daily living in patients with stroke. Mod J Integr Chin Western Med. 2022;31:929–32.

[36] Wang AJ Jin Y Lin L. To investigate the effect of scalp acupuncture combined with mirror therapy on lower limb motor function in stroke patients with hemiplegia. Chin J Phys Med Rehabil. 2022;44:135–7.

[37] Ge C Yin ZL Meng ZX. To investigate the effect of scalp acupuncture combined with mirror therapy on lower limb motor function and walking ability in patients with ischemic stroke. Shanghai J Acupunct Moxibustion. 2021;40:647–51.

[38] Chen LX Li CJ Wang TT. To explore the effect of scalp acupuncture combined with mirror therapy on motor function and living ability of patients with lower limb dysfunction after stroke. Shanghai J Acupunct Moxibustion. 2021;40:279–83.

[39] Cui SY Xu MZ Wang SH. To investigate the effect of acupuncture combined with mirror therapy on lower limb dysfunction in patients with hemiplegia after cerebral infarction. Shanghai J Acupunct Moxibustion. 2017;36:9–13.

[40] Zhang XH Liang JH Huang RM. To observe the curative effect and nursing effect of acupuncture combined with mirror therapy on the recovery of lower limb function after stroke. Sichuan Tradit Chin Med. 2018;36:208–10.

[41] Yang Q Ni L. To explore the rehabilitation effect of acupuncture combined with mirror feedback therapy on lower limb spasticity in stroke patients. World Tradit Chin Med. 2020;15:2983–7.

[42] Luo Q Lv J. To investigate the effect of warm acupuncture combined with mirror therapy on limb function and balance ability in patients with hemiplegia after stroke. Clin Med Res Pract. 2020;5:137–9.

[43] Zhu D ZHU KY Wang LX. To observe the effect of mirror therapy combined with penetrating needling at head point on the rehabilitation of lower limb function in patients after stroke. Chin J Rehabil Med. 2019;34:533–8.

[44] Wang Y Zhang Y Chang YX. To investigate the effect of acupuncture at governor vessel acupoints combined with mirror neuron rehabilitation training on the coordination function of upper limb flexor spasticity, arterial blood flow velocity and the expression of GDF-15 and Trk B proteins after stroke. J Liaoning Univ Tradit Chin Med. 2022;24:154–8.

[45] Wu JS Wang JH Sheng F Wen JD. Effect of acupuncture combined with mirror neuron rehabilitation training on hand function and activities of daily living in patients with hand motor dysfunction after stroke. J Pract Chin Med. 2022;38:831–3.

[46] Mehanna R Jankovic J. Movement disorders in cerebrovascular disease. Lancet Neurol. 2013;12:597–608. 23602779 10.1016/S1474-4422(13)70057-7

[47] Netravathi M Pal PK Indira Devi B. A clinical profile of 103 patients with secondary movement disorders: correlation of etiology with phenomenology. Eur J Neurol. 2012;19:226–33. 21777351 10.1111/j.1468-1331.2011.03469.x

[48] Griffis JC Metcalf NV Corbetta M Shulman GL. Structural disconnections explain brain network dysfunction after stroke. Cell Rep. 2019;28:2527–40.e9. 31484066 10.1016/j.celrep.2019.07.100 PMC7032047

[49] Lee JH Kyeong S Kang H Kim DH. Structural and functional connectivity correlates with motor impairment in chronic supratentorial stroke: a multimodal magnetic resonance imaging study. Neuroreport. 2019;30:526–31. 30932970 10.1097/WNR.0000000000001247

[50] Kwakkel G Kollen BJ van der Grond J Prevo AJ. Probability of regaining dexterity in the flaccid upper limb: impact of severity of paresis and time since onset in acute stroke. Stroke. 2003;34:2181–6. 12907818 10.1161/01.STR.0000087172.16305.CD

[51] Yang A Wu HM Tang JL Xu L Yang M Liu GJ. Acupuncture for stroke rehabilitation. Cochrane Database Syst Rev. 2016;2016:CD004131. 27562656 10.1002/14651858.CD004131.pub3 PMC6464684

[52] Chavez LM Huang SS MacDonald I Lin JG Lee YC Chen YH. Mechanisms of acupuncture therapy in ischemic stroke rehabilitation: a literature review of basic studies. Int J Mol Sci. 2017;18:2270. 29143805 10.3390/ijms18112270 PMC5713240

[53] Radak D Katsiki N Resanovic I. Apoptosis and acute brain ischemia in ischemic stroke. Curr Vasc Pharmacol. 2017;15:115–22. 27823556 10.2174/1570161115666161104095522

[54] Sun Y Xue SA Zuo Z. Acupuncture therapy on apoplectic aphasia rehabilitation. J Tradit Chin Med. 2012;32:314–21. 23297549 10.1016/s0254-6272(13)60031-x

[55] Yang L Tan JY Ma H. Warm-needle moxibustion for spasticity after stroke: a systematic review of randomized controlled trials. Int J Nurs Stud. 2018;82:129–38. 29631145 10.1016/j.ijnurstu.2018.03.013

[56] Zhang B Han Y Huang X. Acupuncture is effective in improving functional communication in post-stroke aphasia: a systematic review and meta-analysis of randomized controlled trials. Wien Klin Wochenschr. 2019;131:221–32. 10.1007/s00508-019-1478-5 31001680

[57] Jan S Arsh A Darain H Gul S. A randomized control trial comparing the effects of motor relearning programme and mirror therapy for improving upper limb motor functions in stroke patients. J Pak Med Assoc. 2019;69:1242–5. 31511706

[58] Zheng Q. Chinese Society of Neurology, Guidelines for early rehabilitation of stroke in China. Chin J Neurol. 2017;50:405–12.

